# Extrinsic and Existential Mortality Risk in Reproductive Decision-Making: Examining the Effects of COVID-19 Experience and Climate Change Beliefs

**DOI:** 10.3389/fpsyg.2021.644600

**Published:** 2021-06-11

**Authors:** David S. Gordon

**Affiliations:** School of Psychology, University of Chester, Chester, United Kingdom

**Keywords:** mortality, life-history theory, existential risk, COVID-19, climate change

## Abstract

While the COVID-19 pandemic has presented an immediate risk to human life around the world, climate change poses an arguably greater—although less immediate—threat to our species’ survival. Within the framework of life-history theory (LHT), this pre-registered study investigated whether extrinsic risk (i.e., external factors that pose a risk to an individual’s life, e.g., COVID-19) and existential risk (i.e., risks with outcomes that threaten the existence of humans as a species, e.g., climate change) had similar or different relationships with reproductive decision-making. A UK representative sample of 325 participants between 18 and 35 years of age was asked to indicate their ideal number of children, ideal age to start having children, and whether their desire for a child had recently changed. Participants were asked about their experiences of COVID-19 and given a series of scales with which to assess their beliefs about climate change. In support of LHT, the study found evidence that knowing people who had been hospitalized with or died of COVID-19 was associated with a greater ideal number of children. Conversely, there was no clear evidence of a relationship between climate change beliefs and reproductive decision-making. The repercussions for understanding how we interpret and respond to different forms of mortality risk are discussed.

## Introduction

Typically, humans desire to have offspring. However, the psychological mechanisms that affect the desire to have children (and the number thereof) are still very much under debate (Sear et al., 2016). One of the more comprehensive approaches taken to addressing this question has been life history theory (LHT). Initially developed to explain between-species differences in reproductive rates, it has since been used to explain within-species variation in reproduction, behavior, and cognition (Scheiner, 1992; Ellis et al., 2009; Menie et al., 2021). LHT posits that because resources are finite, to maximize reproductive success, organisms must make trade-offs in resource allocation depending on the environment: Resources allocated to individual growth (whether physical, mental, or social) cannot also be allocated to the production of offspring and vice versa (Scheiner, 1992; Sear, 2020). Put simply, if the environment is harsh or unstable, it is “best” to reproduce as soon as and as often as possible; however, if the environment is bountiful and stable, then growth should be prioritized.

Over the past 20 years, LHT has been increasingly applied to human behavior and has been used to understand the covariation in many human traits (Luoto et al., 2019a; Ellis et al., 2020; Sear, 2020). A key focus of this research has been to ascertain whether environmental factors trigger fast or slow life histories. That is, research has sought to determine how cues in the local environment that signal harshness and/or unpredictability trigger suites of present- or future-focused behaviors. Fast life-history strategies emphasize present-focused behavior, while slow strategies emphasize future-focused behavior (Nettle and Frankenhuis, 2019). While there is certainly evidence that heritability and genetics affect the development of life-history strategies (see Figueredo et al., 2006), research has often focused on socioeconomic status (SES), particularly but not exclusively during one’s childhood. SES strongly impacts the quality of one’s environment in general (Pepper and Nettle, 2017; Ellis et al., 2020), and SES is used as a proxy for a harsh environment (Griskevicius et al., 2011) and an environment of high morbidity and mortality (Ellis et al., 2009), while other measures have been used to measure environmental unpredictability in childhood (Young et al., 2020). The childhood environment is key to development (Figueredo et al., 2006), and researchers have suggested that cues of environmental quality in early childhood calibrate an individual’s behavioral profile for what is likely a lifetime in that environment (Ellis et al., 2009, 2020; Del Giudice et al., 2015). Childhood SES has been associated with performance in future discounting tasks (Mittal and Griskevicius, 2014; cf., Ellis et al., 2020), personality development (Međedović, 2019), and reproductive decision-making (Griskevicius et al., 2011; Chipman and Morrison, 2015; Jaadla et al., 2020). Nevertheless, the potential for a large mismatch between an individual’s current environment and their childhood environment means that human behavior should also be open to influence throughout one’s life. Indeed, there is also ample evidence that the adult environment also adjusts behavior to a faster or slower approach to life (Quinlan, 2010; Nettle, 2017; Pepper and Nettle, 2017).

### Extrinsic and Existential Risk

One direct and evolutionarily salient cue of a current harsh (or unstable; see Young et al., 2020) environment is death, or rather, a high local mortality rate. Extrinsic mortality risk, which is the risk of mortality over which one has no control, has been shown to increase the speed of one’s life history across numerous domains, including reproductive decision-making (McAllister et al., 2016; Pepper and Nettle, 2017; André and Rousset, 2020). Importantly, such changes in response to extrinsic risk occur in response to the current local environment rather than to the childhood environment alone. For example, high local infant mortality has been associated with an earlier onset of reproduction (Quinlan, 2010), as has the number of bereavements an individual has experienced in the recent past (Pepper and Nettle, 2013). Thus, while childhood experiences likely determine general behavioral tendencies (Mittal and Griskevicius, 2014; cf., Wu et al., 2020), there is likely still plasticity in how one’s life-history strategy calibrates to the current environment (Ellis et al., 2009; Nettle and Bateson, 2015; McAllister et al., 2016). We should expect that any sudden change in extrinsic risk, such as a global pandemic, might impact decision-making.

However, we as a species are unique in that we can comprehend, and cause, a very different type of risk—existential risk. Existential risk refers to threats that could cause human extinction or the permanent curtailing of human progress due to the destruction of the Earth’s potential to sustain life (Bostrom, 2002; Cotton-Barratt and Ord, 2015; Schubert et al., 2019). Such threats could be natural (e.g., an asteroid colliding with Earth), but human technological progress has dramatically increased the threat of artificially inducing such an event (e.g., through rising temperatures as a result of burning fossil fuels). To quote E. O. Wilson, “the real problem of humanity is the following: we have paleolithic emotions; medieval institutions; and God-like technology.”

It could be argued that existential risk is a form of extrinsic risk in that an existential risk is also a personally unavoidable risk to oneself and therefore to one’s future offspring. Thus, the effects of an existential risk on life-history strategy might be no different than those of other cues that indicate potential future hardship (e.g., Wisman and Goldenberg, 2005; Griskevicius et al., 2011). However, by definition, we as a species have not experienced an existential threat (i.e., there are no day-to-day experiences that could act as an evolutionarily salient cue). Indeed, as the creation (e.g., mutually assured destruction) and comprehension (e.g., extraterrestrial objects) of such risks have depended on human technological progress, their evolutionary novelty potentially means they might produce unpredictable behavioral changes.

One example of an existential risk is climate change. While the firsthand effects of climate change are becoming apparent even in societies that have so far been insulated from them (Filkov et al., 2020; Parry et al., 2020), recent evidence suggests that such events are being conceptualized as local extrinsic risks to life rather than manifestations of existential risk (Schubert et al., 2019). Interestingly, while academic research on the psychological consequences of existential risk relevant to LHT is sparse (see Schneider-Mayerson and Leong, 2020), several recent polls have suggested that concerns regarding climate change are reducing the desire to have children (Miller, 2018; Relman and Hickey, 2019). The results of this polling data were recently supported by Schneider-Mayerson and Leong (2020); however, the results are at odds with the principles of LHT. If an individual fears greater future instability, should this fear not lead to a fast life history and a desire for more children now, while resources (at least in the West) are still plentiful? Indeed, with priming experiments demonstrating that imagining a harsh or unstable future leads to a faster life history (see McAllister et al., 2016), one would expect the same response in those that heed the warnings of climate scientists. As stated, the evolutionary novelty of an existential risk might result in unexpected behavior changes due to the mismatch between current and ancestral conditions (see Li et al., 2018): Visible cues pointing to the precariousness of one’s own mortality in the local environment might produce different behavioral responses when compared to more abstract concerns regarding future species-wide mortality due to large-scale ecological collapse (generated, for example, by exposure to media; see Brulle et al., 2012; Dunn et al., 2020).

### The Current Study

The COVID-19 pandemic provides the opportunity to study both extrinsic and existential risks simultaneously. COVID-19 has increased the extrinsic mortality risk in Western, Educated, Industrialized, Rich, and Democratic (WEIRD) societies in a manner arguably not seen since the end of World War II. Therefore, it provides a novel opportunity to assess the impact of a sudden change in mortality risk on reproductive decision-making in a WEIRD population (i.e., the United Kingdom). It also allows for a comparison to be made between a sudden rise in extrinsic mortality risk and the existential risk posed by climate change. In doing so, it adds to the dearth of information regarding the effects of the latter.

The current study investigated whether experiences of COVID-19 and climate change beliefs impacted reproductive decision-making. Participants were asked to indicate their ideal number of children and the ideal age at which to have the first child. They were then explicitly asked whether their desire for a child or another child had increased or decreased during the pandemic. As stated in the pre-registration, it was predicted that COVID-19 experience (measured by illness experienced by the participant and their close associates and deaths of the latter) would predict an increase in the ideal number of children and a decrease in the ideal age of first birth. It was also predicted that COVID-19 experience would predict an increase in the immediate desire for a child or another child. In terms of climate change, no directional prediction was made. Available evidence from polling has suggested that climate change concerns should curtail reproduction,^1^ whereas LHT and priming studies within the latter theoretical framework have suggested that the opposite pattern should occur.

## Materials and Methods

### Participants

Using the online platform Prolific (Damer and Bradley, 2014),^2^ a statistically representative sample of the UK population aged between 18 and 35 years was recruited. Using G*Power (Erdfelder et al., 1996), the sample size was determined with an anticipated “small effect” with 10 predictors (power = 0.95). Three hundred and twenty-five participants completed the online survey, but 26 declined to answer the COVID-19 experience questions and were removed from the study. Of the remaining 297 participants, 245 identified as White, 27 as Asian, 9 as Black, 9 as of mixed heritage, and 7 as “other.” One hundred and fifty-seven participants were female. Participants were paid £3 for completing the survey. Data collection took place at the beginning of August 2020, around 4 or 5 months after the first UK national lockdown was declared in response to the COVID-19 pandemic.

### Outcome Variables

The study measured three outcome variables relating to reproductive decision-making. Participants were asked to report their ideal number of children and what would be the ideal age at which to have their first child. For the third outcome variable, participants were asked whether the COVID-19 pandemic had affected their desire to have a child or another child on a scale of −3 (*much less desire*) to +3 (*much more desire*), which was coded 1–7 for analysis purposes and labeled “change in desire.” Participants who did not want children were able to indicate this.

### Predictor Variables

The extrinsic threat from COVID-19 was operationalized as the participant’s reported experience with the illness. As per the pre-registration, there were two variables of interest. The first was whether participants believed they had caught COVID-19 (regardless of whether they had received a confirmed positive test result) and the degree of their symptoms: no or “don’t believe so,” mild symptoms, moderate symptoms, severe symptoms without hospital admission, or required hospitalization. A list of symptoms for each degree of severity was provided. Participants were then asked to indicate how many people close to them had experienced a (suspected) COVID-19 infection, including how many people had died or been hospitalized.

Existential threat was measured by views on climate change. Three predictor variables were collected. To measure “worry” about climate change, participants were asked Question 4 from the latest RESiL RISK survey of climate change attitudes (Steentjes et al., 2020): “How worried, if at all, are you about climate change?” Their answers were assessed using a scale from 1 (*not worried at all*) to 5 (*extremely worried*).

To measure “expectations” of climate change, participants responded to Question 17 of the same survey. This question was comprised of 16 items that asked participants to indicate on a scale of 1 (*very unlikely*) to 5 (*very likely*) how likely various outcomes of climate change were to occur in the United Kingdom (e.g., “Cities and large towns, which trap heat, becoming unbearably hot due to heatwaves”; *α* = 0.91).

Participants were also assessed on their level of climate denialism using the dominance and climate change denial scale (Häkkinen and Akrami, 2014). While listed in the pre-registration, this questionnaire was excluded from the analysis because of low reliability (*α* = 0.55).

### Covariates

To discern whether COVID-19 experience and climate change beliefs impact reproductive decision-making, several covariate variables were collected to act as controls in the General Linear Model. Participants were asked how many people they felt close to had died in the past 5 years prior to January 2020. Participants were also asked to indicate their perceived SES using the MacArthur Scale of Subjective Social Status (Singh-Manoux et al., 2003). This measure asks participants to imagine society as a ladder where those at the top have the best jobs, the most money, and the most opportunities and those at the bottom have the least (1 = *the very bottom* to 10 = *the very top*). Participants indicated their place on the ladder based on their current situation and their childhood situation. Participant age was also recorded.

### Procedure

To ensure the COVID-19 and climate change questions did not influence participant responses to the critical reproductive decision-making questions, the survey was given in a specific order and all participants experienced the same order (although the arrangement of individual items was randomized). To further prevent the true nature of the study from being discerned, distractor items and questionnaires were also included.

Participants were first asked the three reproductive decision-making questions (see section “Predictor Variables”). To prevent the true nature of the study from being discerned, participants were also asked similar questions regarding home ownership, business ownership, large purchases (over £500), and retirement plans. Participants then completed the mini IPIP (Donnellan et al., 2006), which was the first of two distractor questionnaires,^3^ followed by the “worry” and “expectation” climate change measures. Participants then completed the second distractor questionnaire, The Dirty Dozen (a short measure of the Dark Triad; Jonason and Webster, 2010), followed by the climate change denial scale. Participants then indicated how many of their close associates had died in the 5 years prior to January 2020, and then, they answered the COVID-19 questions. Finally, participants indicated their current SES and childhood SES.

### Statistical Analysis

A GLM was used to investigate whether the COVID-19 experience and climate change belief variables predicted reproductive decision-making; the alpha threshold was set at *p* < 0.017 because there were three separate outcome variables. For all pre-registered analyses, age, sex, and both childhood and current SES were entered into the model as controls. While not predicted in the pre-registration, exploratory analyses were carried out to investigate whether any effects of extrinsic and existential risk on reproductive decision-making were moderated by childhood SES. Moderation analyses were conducted using PROCESS (Hayes, 2012). All analyses were conducted in SPSS 26.

### Ethics

This study was conducted with the full ethical approval of the School of Psychology’s (University of Chester) Research Ethics Committee. Participants gave written informed consent before taking part in the study; they were fully debriefed once their participation was complete and given the option to withdraw their data without penalty should they desire.

## Results

Sixty-seven participants already had children and 230 did not; no *a priori* assumption was made that the former would affect either “change in desire” or “ideal number of children,” so they were included in those analyses. However, they were removed from the “ideal age” question since logically, COVID-19 could not have affected their decision. Of those who did not already have children, 36 participants indicated that they did not want children. Since no *a priori* assumption was made regarding those who wanted to remain childless (their decision could have been related to COVID-19, climate change, or some unrelated factor), they were included in the change in desire and ideal number of children analyses. However, they were excluded from the question regarding ideal age. Thus, all 297 participants were entered into the analyses of ideal number of children and change in desire for a child, and 196 participants were entered into the analysis of ideal age.^4^

The nature of the data required some departure from the pre-registered variables. Two hundred and nineteen participants reported not becoming sick from COVID-19, 49 believed they had experienced mild sickness, 24 believed they had experienced moderate sickness, three believed they had experienced severe symptoms, and two preferred not to say. As a result, the variable “own sickness” was converted into a dichotomous variable indicating whether the participant had become sick (*N* = 78) or not. Equally, only 49 participants indicated they knew at least one person who had either required hospital treatment or had died due to COVID-19. This variable was also converted into a dichotomous variable, labeled “other sickness.” No other changes were made. Descriptive statistics for the continuous variables can be found in Table 1.

**Table 1:** Descriptive statistics.

	Age	Ideal age^1^	Ideal number of children	Change in desire	Climate worry	Climate expectation	Bereavements	Current SES	Childhood SES
*M*	26.7	29.79	2.10	4.16	3.28	3.82	1.41	5.13	5.18
*SD*	5.16	3.38	1.17	1.32	1.02	0.65	1.52	1.69	1.89
Min.	18	22	0	1	1	1	0	1	1
Max.	35	40	7	7	5	5	8	9	10

1 *Excluding participants who already had children or who do not wish to have children (N = 196)*.

### Ideal Number of Children

Evidence showed that experience with COVID-19 was associated with ideal number of children. As shown in the summary of the full model containing all predictors (Table 2), with all predictors entered, other sickness significantly predicted ideal number of children, with those who knew someone who had been hospitalized or died from COVID-19 reporting a greater number of children as ideal (Adj. *R*^2^ = 0.06, *F*_9,287_ = 2.19, *p* = 0.02). However, following the recommendations of Achen (2005; see also Corpuz et al., 2020), the effect of each predictor was investigated separately along with the control variables. The only significant predictor of ideal number of children was whether participants knew someone who had become seriously ill with COVID-19 [other sickness: $R_{\mathrm{Adjusted}}^{2}$ = 0.06, *F*_6,290_ = 3.07, *p* = 0.006; *b* = 0.42, *SE* = 0.18, BCa 95% CI (0.07/0.76), *p* = 0.016]. The latter model can be considered the most parsimonious and falls under our conservative threshold for significance, although this approach was not specified in the pre-registration. There was no evidence that climate change belief was associated with ideal number of children.

**Table 2:** Summary of coefficients for full models.

	Ideal number of children			Change in desire for children		
	*b* (se)	BCa 95% CI	*p*	*b* (se)	BCa 95% CI	*p*
*Constant*	*3.15 (0.54)*	*2.08/4.20*	*<0.001*	*3.23 (0.78)*	*1.70/3.99*	*<0.001*
Own sickness^1^	0.12 (0.14)	−0.14/0.38	0.43	0.03 (0.19)	−0.38/0.40	0.86
Extreme exposure^2^	**0.39 (0.17)**	**0.07/0.72**	**0.02**	0.12 (0.25)	−0.39/0.57	0.60
Climate worry	0.03 (0.07)	−0.10/0.17	0.64	−0.16 (0.11)	−0.35/0.06	0.14
Climate expectation	−0.10 (0.11)	−0.32/0.13	0.37	**0.37 (0.17)**	**0.05/0.67**	**0.029**

*Bereavement, age, sex, childhood SES, and current SES were held constant in all analyses. Bias-corrected 95% CI bootstrapped (5,000 sample). Reference category* =

1 *Had not contracted COVID;*

2 *Did not know anyone who had been hospitalized with or died of COVID-19*. Bold indicates p < 0.05.

### Change in Desire for a (Another) Child

There was no clear evidence that any of the predictor variables were associated with a change in the desire for a child. As shown in Table 2, in the full model, the “climate expectation” variable did show a significant relationship with change in desire, with a greater expectation of negative consequences from climate change predicting an increase in the recent desire to have children. However, there was no overall significant model fit ($R_{\mathrm{Adjusted}}^{2}$ = 0.01, *F*_9,287_ = 1.19, *p* = 0.30). Equally, when each predictor was entered individually into a model with the control variables, none were significant. Thus, no predicted variable can be claimed to have had a relationship to change in desire.

### Ideal Age for First Child

No measures of COVID-19 experience or climate change belief were associated with the ideal age to have children. However, there was a strong positive correlation between participant age (minus the exclusions, *M* = 25, *SD* = 5, Min. = 18, Max. = 35) and ideal age to have a first child [*r* = 0.56, *N* = 196, *p* < 0.001, BCa 95% CI (0.45/0.66)]. In the model and moderation analyses, participant age was the sole predictor of ideal age (*p* < 0.001).

### Exploratory Analysis: Moderating Effects of Childhood SES

The primary aim of the current study was to investigate extrinsic and existential risk based on whether cues of risk (i.e., severe COVID-19 infection in oneself or one’s associates) and beliefs about climate change were associated with reproductive decision-making. Nevertheless, given the established association between risk and early life stress, exploratory analyses were also conducted to investigate whether such effects were present in the data. As the study cannot be considered appropriately powered to detect such interactions (Achen, 2005; Giner-Sorolla, 2018), the results should be interpreted with caution.

Table 3 shows GLMs with each individual variable and controls and an interaction between childhood SES and the variable. As shown in Table 3, the relationship between other sickness and ideal number of children was not moderated by childhood SES, with other sickness remaining a significant predictor of ideal number of children with the interaction included in the model. Childhood SES did not moderate the relationship between own sickness and ideal number of children (Table 3).

**Table 3:** Moderating effect of childhood SES on ideal number of children.

Models		*R*^2^	*F* (df)	*p*	Boot *b* (se)	Boot 95% CI
1.	Own sickness					
	*Constant*	**0.06**	**2.49 (7,289)**	**0.017**	*2.83 (0.37)*	*2.10/3.54*
	Own sickness^1^				0.18 (0.14)	−0.09/0.46
	Childhood SES				−0.01 (0.04)	−0.07/0.09
	C.SES*own sickness				−0.15 (0.08)	−0.31/0.007
2.	Other sickness					
	*Constant*	**0.06**	**2.69 (7,289)**	**0.01**	*2.79 (0.37)*	*2.05/3.51*
	Other sickness^2^				**0.40 (0.17)**	**0.06/0.74**
	Childhood SES				−0.01 (0.04)	−0.09/0.07
	C.SES*extreme exposure				−0.05 (0.10)	−0.26/0.13
3.	Climate worry					
	*Constant*	**0.05**	**2.36 (7,289)**	**0.02**	*2.87 (0.38)*	*2.16/3.58*
	Climate worry				0.01 (0.06)	−0.10/0.15
	Childhood SES				−0.02 (0.04)	−0.10/0.05
	C.SES*climate worry				**0.07 (0.03)**	**0.00/0.14**
4.	Climate expectation					
	*Constant*	0.05	2.02 (7,289)	0.052	*2.89 (0.40)*	*2.10/3.68*
	Climate expectation				−0.06 (0.10)	−0.24/0.13
	Childhood SES				−0.03 (0.04)	−0.10/0.05
	C.SES*climate expectation				0.07 (0.05)	−0.30/0.16

*Bereavement, age, sex, and current SES were held constant in each analysis. All non-dichotomous variables were mean centered and bootstrapped (5,000 sample). Reference category* =

1 *Had not contracted COVID-19;*

2 *Did not know anyone who had been hospitalized with or died of COVID-19*. Bold indicates p < 0.05.

**Figure 1:**
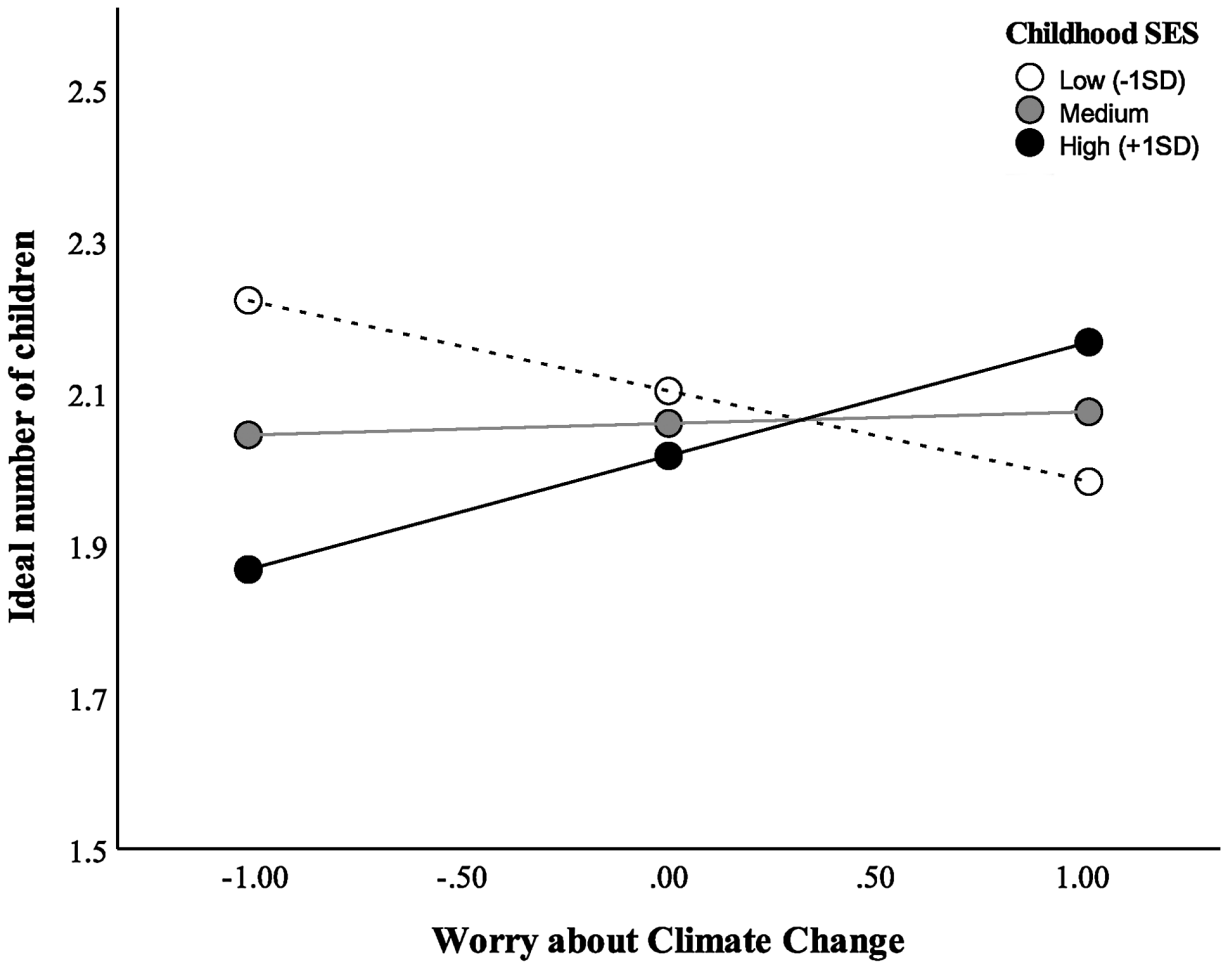
Relationship between ideal number of children and worry about climate change, moderated by childhood SES.

As shown in Table 3 and Figure 1, childhood SES moderated the relationship between climate worry and ideal number of children. However, a simple slopes analysis showed there was no relationship between climate worry and ideal number of children at high, average, or low levels of childhood SES (*p* > 0.05). Using the Johnson–Neyman (J–N) technique to further probe for sensitivity (Hayes and Matthes, 2009), the J–N point for *p* < 0.05 of childhood SES occurred at −4.32 and +3.82 of the mean. The region of significance contained 2% of the sample.

No moderating effects were found for the reproductive decision-making variables ideal age or change in desire.

## Discussion

The current study investigated the impact of extrinsic and existential mortality risk on reproductive decision-making in a WEIRD population. In line with LHT, it was predicted that COVID-19 experience (extrinsic risk) would be associated with responses indicative of a faster life history, with greater experience being associated with a greater ideal number of children, a lower ideal age at which to have children, and a recent increase in the desire to have a child or another child. It was also predicted that beliefs about climate change (existential risk) would be associated with reproductive decision-making, although no directional predictions were offered due to conflicting evidence (Schneider-Mayerson and Leong, 2020). Some of these predictions were supported.

### Extrinsic Risk and Reproductive Decision-Making (COVID-19 Experience)

The results suggested that being close to someone who was seriously ill or died from COVID-19 was associated with a greater ideal number of children. This association supports both the prediction of the study and previous research on the role of life history in reproductive decision-making. Research has shown that local cues of mortality risk affect reproductive decision-making (Quinlan, 2010; Pepper and Nettle, 2013; McAllister et al., 2016), and knowing individuals who were seriously ill or died from COVID-19 serves as a cue of an elevated risk of mortality in the environment. As such, the result suggests that even a brief change in extrinsic risk can potentially result in a faster life history. For obvious reasons, the experimental literature on the effect of mortality risk has relied on primed cues of mortality (Wisman and Goldenberg, 2005; Mathews and Sear, 2008; Griskevicius et al., 2011), thus limiting the ecological validity of the findings. The current study supports the conclusions of these studies by demonstrating the relationship between an actual sudden change in extrinsic mortality risk (i.e., the COVID-19 pandemic) and reproductive decision-making. More broadly, the results also provide further empirical support for adult changes in life history due to changes in extrinsic risk (Pepper and Nettle, 2013).

There has been some debate as to what exactly acts as a cue to mortality risk (Pepper and Nettle, 2014; Chipman and Morrison, 2015; Wu et al., 2020). Research thus far has suggested that participants understand that COVID-19 poses a real risk to health (see Sutton and Douglas, 2020). As such, it is interesting that COVID-19 experience predicted reproductive decision-making—or rather, that the dramatic change in everyday life and the daily reported death toll did not raise the floor of responses sufficiently for no effect to be detected. Conversely, at the time of data collection in early August 2020, the United Kingdom was at the bottom of the first wave of the pandemic, and the focus was on returning to “normal” life (e.g., there was a scheme offering discounts on restaurant meals; see “Eat Out to Help Out,” HM Treasury, 2020), and news of vaccine successes may have given the impression that the crisis would be over soon (University of Oxford, 2020). Both factors could have lowered the ceiling on the perceived current and future risk from COVID-19. The results therefore highlight that despite a media environment saturated with COVID-19 information, a change in life history was associated with a close experience of mortality cues.

While the study used COVID-19 infection to operationalize extrinsic risk, susceptibility to disease is also an intrinsic risk. Intrinsic risk—meaning risks dependent on internal factors or personal behavior—has also been shown to impact life-history strategy. For example, MHC homozygosity (Murray et al., 2017) and a history of vulnerability to illness (Hill et al., 2015) have been linked with a faster life history, and some research has associated immunocompetence with childhood SES (Rubika et al., 2020). Equally, Corpuz et al. (2020) found that a fast life history was associated with less engagement with and endorsement of health advice. As such, we may have expected childhood SES—a factor predictive of a faster life history (e.g., Griskevicius et al., 2011)—to have moderated the relationship between the COVID-19 variables and reproductive decision-making. Such a relationship was not found, but this may have been due to the lack of statistical power to detect interaction effects. A higher-powered examination of how COVID-19 infection might differentially affect those with a faster lift history is certainly warranted.

No relationship was found between COVID-19 experience and the other outcome variables regarding the ideal age at which to have the first child or whether the desire for a child had changed since the beginning of the pandemic. For the latter, it does appear contradictory that COVID-19 exposure would predict an increase in the ideal number of children but not in the reported change in desire. The reason may be due to the construction of the question. Most studies have asked participants about their general future desire (Wisman and Goldenberg, 2005; Griskevicius et al., 2011), whereas the question created for the current study asked participants to think very specifically about their current circumstances. Thus, the results might represent a difference between generality and specificity when eliciting responses: Asking participants to “think about the last 6 months” required a more specific examination of their immediate situation compared to the question “what is your ideal number of children?” Importantly, this supposition is supported by the data from previous pandemics where there is a brief reduction in births before a “boom” (Ullah et al., 2020). Thus, asking participants to consider their immediate circumstances produced different responses compared to asking a general question about family planning.

There is a more straightforward explanation for the lack of any associations between COVID-19 experience and ideal age of first birth—COVID-19 is a sudden and very recent event. Past studies examining mortality cues and reproductive decision-making have operated over a larger window in terms of the type of environmental risk studied, and they have typically included younger age groups (Pepper and Nettle, 2013; Häkkinen and Akrami, 2014; Virgo and Sear, 2016). Given the plethora of proximate factors affecting reproductive scheduling in post-demographic transition societies (e.g., Burnside et al., 2012; Sear et al., 2016), it may be that the demographic window of the current sample was too narrow for any effect of COVID-19 on reproductive scheduling to be apparent. Indeed, the mean age at first childbirth in the United Kingdom is 29 years (ONS, 2019), which is not far from the mean age of the participants, and the analyses showed that ideal age was solely predicted by actual age.

Still, the demographics of the study do provide room for COVID-19 experience to influence life history. It was predicted that an increase in extrinsic mortality risk would be associated with all aspects of reproductive decision-making, and the prediction regarding ideal age at first birth was not supported. That only one of the three metrics of reproductive decision-making showed the predicted relationship with COVID-19 experience should be taken into consideration when drawing any conclusions about the impact of COVID-19 on life-history strategies. Nevertheless, with other factors that affect life history controlled for, knowing others who had become seriously ill with COVID-19 was related to the number of children the participants desired, even if it was not related to the age at which they planned to start.

### Existential Risk and Reproductive Decision-Making (Climate Change)

There was no indication that climate change beliefs were associated with reproductive decision-making overall, which would be expected based on the results from previous studies that have primed precarious, although non-specific, futures (McAllister et al., 2016). One of the key tenets of LHT is that the early childhood environment and the immediate environment provide cues to the future to which organisms are sensitive (Del Giudice et al., 2015; Pepper and Nettle, 2017; Nettle and Frankenhuis, 2020). However, recognizing the real possibility of global human extinction is a new phenomenon. The evolutionary novelty of existential risk potentially means that human existence does not influence life-history strategy as we might have logically surmised (Li et al., 2018; Schubert et al., 2019; Young et al., 2020). Indeed, the exploratory moderation analyses suggested that the moderating effects of childhood SES on the relationship between the ideal number of children and worry about climate change ran counter to what would be expected according to LHT.

The reproductive decision-making responses to the very salient and (at the time of writing) ongoing COVID-19 pandemic were predicted by direct experience. However, worrying about future outcomes of climate change is very different from worrying about a measure of local violent crime or being asked to imagine one’s own death, for example. Thus, due to their novelty, concerns about existential risks might simply not act as reliable cues of future or present instability as imagined by LHT (Young et al., 2020). Indeed, evidence from a US sample suggested that the experience of extreme weather events was not reflected in concerns about climate change (Brulle et al., 2012). Instead, any response to more abstract existential thoughts (potentially “slow” thoughts; Kahneman, 2011) about the future might be better set in the context of resource allocation in post-demographic transition societies (e.g., Burnside et al., 2012; Sear et al., 2016). Interestingly, while it has been suggested that existential risk is an evolutionary novelty, the moderation by childhood SES observed in this study (albeit for only one of the outcome variables) is potentially similar to historical patterns of reproductive decision-making in resource-limited environments (Volk, 2021). The study is not able to address this debate further, but it is a question certainly worth exploring.

The lack of any concrete results regarding climate change and reproductive decision-making does contradict the little evidence that exists on this topic (Schneider-Mayerson and Leong, 2020). This is likely due to methodological differences, as Schneider-Mayerson and Leong (2020) recruited participants through a largely US-based activist network and emphasized policy issues (e.g., the carbon footprint of a child). Unsurprisingly, that sample reported higher climate change concerns than the sample in the current study and compared to the UK population in general (see Steentjes et al., 2020). Thus, if there is one conclusion that can be drawn from the climate change data, it should be that the reports of climate change concerns reducing the desire to have children are perhaps premature.

### Future Directions

While the study aimed to investigate the relationship between extrinsic and existential risk and reproductive decision-making, the measures of each were very different. Extrinsic risk was operationalized as COVID-19 experience, whereas existential risk was measured through attitudinal questionnaires. As such, the current study did not ask about any experiences with climate change (e.g., destruction of property due to flooding). However, once this occurs, climate change arguably becomes an extrinsic mortality risk. Semantic arguments notwithstanding, it should be possible to investigate existential risks without engaging in such a debate. Future cross-sectional research might wish to investigate whether living in environments associated with existential risk cues (e.g., along coastlines or near nuclear weapons facilities) is associated with faster life histories. Finally, while climate change might lead to both a harsh and unpredictable future, the childhood SES measure used in the study can be indicative of a harsh early environment but not necessarily an unstable one (see Young et al., 2020). Measures of early life instability may yield different patterns of interaction between that variable and measures of local mortality (e.g., COVID-19) and climate change beliefs.

Equally, with its cross-sectional design, the current study cannot demonstrate causation. Building on the methodologies of prior work (e.g., Griskevicius et al., 2011), experimental research might investigate whether priming participants with existential risks produces results that are contrary to or in accordance with LHT, such as priming for existential risk in general, for specific possible consequences (e.g., personal property damage or contact with displaced populations, see Vardy and Atkinson, 2019), or to make the risk to future offspring especially salient.

Furthermore, as the results for climate change belief did not correspond to either LHT or the limited sociological data available, it is possible that two forces are acting against one other regarding reproductive decision-making: Existential risk cues might be unconsciously inducing a faster life history, while conscious thought regarding children’s future experiences might be reducing the desire to have them. The current study was not designed to investigate this, but future work examining interactions between existential beliefs and morality cues would certainly help shed further light on the place of existential risk within the LHT framework.

Finally, the current study was conducted using a representative sample of a WEIRD society (i.e., the United Kingdom). Doing so allowed for an investigation of life-history responses to a sudden national increase in mortality risk in a population that had not experienced such a change in generations. Still, a relationship has been found between broader ecological factors, such as climate and pathogen load, and life histories (Luoto et al., 2019b), and any future cross-cultural investigations of the legacy of the pandemic should take such factors into account. It is also important to note that numerous WEIRD and non-WEIRD populations are already experiencing the negative effects of climate change (Dannenberg et al., 2019). Additional research within these populations would also help further address the questions raised by the current study.

### Conclusion

The study investigated the impact of extrinsic mortality risk (experience of the COVID-19 pandemic) and existential risk (beliefs about climate change) on reproductive decision-making in a WEIRD society. In line with LHT, COVID-19 experience was associated with a greater ideal number of children. Beyond providing further empirical support for the utility of LHT in understanding human behavior, the current study provides important practical considerations for any policy response to the COVID-19 pandemic. Over a year into the pandemic, the media (Meredith, 2021) and policymakers (Public Health England, 2021) have focused on its consequences for mental health, but by demonstrating that COVID-19 exposure is associated with reproductive decision-making, the results of this study suggest that COVID-19 experience will have broader implications for a wide range of behaviors associated with life-history strategies. Further research on this topic will be vital in understanding the long-term consequences of the pandemic.

The study did not find any consistent evidence of a relationship between existential risk and reproductive decision-making. Given the urgency of climate change and other existential risks (Bostrom, 2002), additional research is warranted to examine further how evolved responses interact with this form of risk and what form those responses take. This is especially vital to understand since faster life-history strategies will arguably be counter-productive to find any global solutions as the everyday impact of climate change becomes increasingly apparent (i.e., as it becomes an extrinsic risk).

## Data Availability Statement

The datasets presented in this study can be found in online repositories. The names of the repository/repositories and accession number(s) can be found at Open Science Framework. Project title: Extrinsic and existential mortality risk in reproductive decision-making. Available at: https://osf.io/ytx9d/.

## Ethics Statement

The studies involving human participants were reviewed and approved by the School of Psychology’s (University of Chester) Research Ethic Committee. The patients/participants provided their written informed consent to participate in this study.

## Author Contributions

The author confirms being the sole contributor of this work and has approved it for publication.

### Conflict of Interest

The author declares that the research was conducted in the absence of any commercial or financial relationships that could be construed as a potential conflict of interest.

## Footnotes

^1^Schneider-Mayerson and Leong (2020) was published after the current study’s registration and data collection had concluded.

^2^Demographic percentages were computed using Home Office statistics (ONS, 2018) with individual inclusion based on each participant’s Prolific profile.

^3^The mini IPIP (Donnellan et al., 2006) and The Dirty Dozen (Jonason and Webster, 2010) were included to avoid participants recognizing that the study was concerned specifically with beliefs about climate change.

^4^Two individuals who had children indicated they did not want children.
